# Elucidating the Interactions between Influenza Virus Polymerase and Host Factor ANP32A

**DOI:** 10.1128/JVI.01353-19

**Published:** 2020-01-17

**Authors:** Bhakti Mistry, Jason S. Long, Jocelyn Schreyer, Ecco Staller, Raul Yusef Sanchez-David, Wendy S. Barclay

**Affiliations:** a Section of Molecular Virology, Imperial College London, London, United Kingdom; Cornell University

**Keywords:** host factor, influenza, polymerase

## Abstract

The avian-origin influenza A virus polymerase is restricted in human cells. This restriction has been associated with species differences in host factor ANP32A. Avian ANP32A supports the activity of an avian-origin polymerase. However, the avian-origin polymerase is incompatible with human ANP32A. Avian ANP32A proteins harbor an additional 33 amino acids compared to human ANP32A proteins, which are crucial for their ability to support the avian-origin influenza virus polymerase. Here, we elucidate the interactions between ANP32A proteins and the influenza A virus polymerase using split luciferase complementation assays, coimmunoprecipitation, and *in situ* split Venus interaction assays. We show greater interaction of chicken ANP32A than human ANP32A with the viral polymerase and visualize these interactions *in situ* in the cell nucleus. We demonstrate that the 33 amino acids of chicken ANP32A and the PB2 627 domain of viral polymerase complex both contribute to this enhanced interaction. Finally, we show how these interactions are affected by the presence of viral RNA and the processivity of the polymerase, giving insights into the way that ANP32A might act during virus infection.

**IMPORTANCE** Successful zoonotic transmission of influenza A virus into humans can lead to pandemics in an immunologically naive population. Host-encoded ANP32A proteins are required to support influenza A virus polymerase activity, and species differences in ANP32A can restrict the host range of influenza virus. Understanding how ANP32A proteins support the viral polymerase and how differences in ANP32A affect the ability of the polymerase to coopt these proteins will enhance our understanding of viral replication and species restriction as well as suggesting targeted antiviral approaches to treat influenza virus infection.

## Abstract (precis)

Successful zoonotic transmission of influenza A virus into humans can lead to pandemics in an immunologically naive population. Host-encoded ANP32A proteins are required to support influenza A virus polymerase activity, and species differences in ANP32A can restrict the host range of influenza virus. Understanding how ANP32A proteins support the viral polymerase and how differences in ANP32A affect the ability of the polymerase to coopt these proteins will enhance our understanding of viral replication and species restriction as well as suggesting targeted antiviral approaches to treat influenza virus infection.

## INTRODUCTION

Influenza A virus (IAV) naturally resides in wild waterfowl. However, zoonotic transmission into humans can result in pandemics in an immunologically naive population. To be capable of replication and efficient transmission between humans, the virus must undergo adaptions to overcome the host range barriers present in the new host. These barriers exist at several points during the viral replication cycle, including replication of the viral genome by the IAV RNA polymerase (1).

The IAV polymerase consists of three subunits, PB1, PB2, and PA. The heterotrimeric polymerase binds to the negative-sense viral RNA (vRNA) of influenza virus to carry out both transcription and replication of the genome in the nucleus of host cells. Replication is achieved through a positive-sense cRNA intermediate. NP is also required for these functions and, together with the polymerase and RNA, forms a complex known as a viral ribonucleoprotein complex (vRNP).

The most common adaption in the avian-origin viral polymerase, to overcome restriction in mammalian cells, is the E627K mutation on the PB2 subunit of the polymerase (2, 3). Since amino acid 627 of PB2 lies on a solvent-exposed region of the polymerase, it is accessible to host factors, and it is likely that differences in factors in different hosts drive this mutation (4). Several host factors have been implicated in this host switch, including the importins, DDX17, RIG-I, and, most recently, ANP32A (5 – 8).

We previously discovered that avian ANP32A is required for the activity of an avian-origin virus polymerase. Overexpression of chicken ANP32A (chANP32A) in human cells could rescue the function of avian virus polymerase, which otherwise has very little activity in these cells (8). Avian ANP32A differs from its human homologues by an additional 33 amino acids between the LRR and LCAR domains of the protein encoded on an additional exon. The first 6 of the additional amino acids are unique and form a SUMO-interacting motif (SIM) (9). The following 27 amino acids are a partial duplication of the sequence upstream. Human ANP32A and -B (huANP32A and -B, respectively) support the activity of a human-adapted influenza virus polymerase (10 – 13), suggesting that the PB2 E627K mutation may be an adaption toward using the shorter human homologues of ANP32A.

How ANP32A supports the activity of the polymerase remains unclear. Previous work has shown that huANP32A and -B can promote synthesis of vRNA from a cRNA template *in vitro* (10). Interactions between the polymerase and ANP32 have previously been demonstrated using coimmunoprecipitation assays (9, 10, 13 – 15); however, there is conflicting evidence as to whether these interactions are stabilized within RNPs. Baker et al. showed an increase in interaction of the polymerase with chANP32A in the presence of a viral-like RNA (15). On the other hand, Sugiyama et al. were not able to coprecipitate NP with huANP32A or -B from infected-cell lysates, suggesting that huANP32A and -B do not bind to polymerase within RNPs (10).

Here, we developed split luciferase and split Venus complementation assays to characterize interactions between influenza virus polymerase and ANP32A proteins. We demonstrate that these interactions occur in the nucleus. We confirmed that the binding of polymerase to chANP32A is greater than that to huANP32A proteins and that this is mediated by the additional 33 amino acids that interact with the 627 domain of PB2. However, using these assays, we did not measure a significant increase in the interaction between human ANP32A and viral polymerase bearing the E627K PB2 adaptation, suggesting that increased interaction does not entirely explain how this mutation determines the host range of influenza virus. We find that binding of ANP32A to polymerase is stabilized in the presence of viral RNA when polymerase is inactive, but the interaction is decreased under conditions where polymerase replicates, altogether providing further insight into the mechanisms by which ANP32 proteins can support polymerase activity.

## RESULTS

### Influenza virus polymerase interacts with ANP32A proteins.

**FIG 1:**
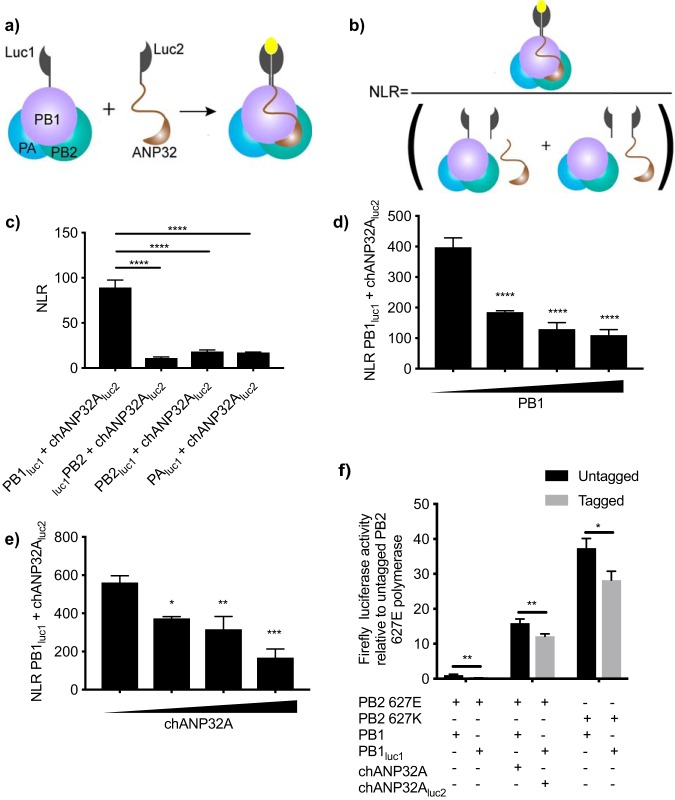
Development of split luciferase complementation assay. (a) Schematic of split luciferase complementation assay. Interaction of putative interacting partners reconstitutes *Gaussia* luciferase, resulting in luminescence upon addition of substrate. (b) Calculation of normalized luminescence ratio (NLR). (c) luc-tagged polymerase components were transfected into HEK 293T cells along with chANP32A_luc1_ and the remaining polymerase components. (d and e) HEK 293T cells were transfected with expression plasmids encoding PB1_luc1_, chANP32A_luc2_, PB2 627E, PA, and 0, 6.25, 12.5, or 25 ng untagged PB1 (d) or untagged chANP32A (e). (f) Minigenome components were transfected into HEK 293T cells including either untagged PB1 or PB1_luc1_ and untagged chANP32A or chANP32A_luc2_. Total DNA was kept constant between samples by addition of an empty vector. Results presented relative to untagged PB2 627E polymerase activity (leftmost black bar). Twenty-four hours after transfection, cells were lysed and luminescence was measured. Results shown are mean ± standard deviation from triplicate samples. Results representative of three independent experiments. Statistical significance was assessed by one-way analysis of variance, and comparisons were made between each condition in panel c and to the sample with 0 ng additional PB1 or chANP32A (leftmost black bar) in panels d and e. Multiple *t* tests were carried out for panel f. *, *P* < 0.05; **, *P* < 0.01; ***, *P* < 0.001; ****, *P* < 0.0001. Nonsignificant comparisons have not been annotated.

In order to quantify interactions between ANP32A and the influenza virus polymerase, we developed a split luciferase complementation assay. Residues 18 to 109 of *Gaussia* luciferase were fused onto a component of the viral polymerase, and residues 110 to 185 were fused onto ANP32A. Interaction of the two proteins results in reconstitution of a functional *Gaussia* luciferase enzyme, the activity of which is then measured by addition of substrate (Fig. 1a). Normalized luminescence ratios were calculated for each sample to show the specificity of the interaction over background (Fig. 1b). We chose to develop the assay using the construct that fused the N terminus of *Gaussia* luciferase onto the C terminus of PB1 and the C terminus of *Gaussia* luciferase onto the C terminus of chANP32A, since this combination gave the highest luciferase signal, likely because it allowed a sterically optimal alignment of the luciferase fragments (Fig. 1c). In these experiments, all three components of the polymerase of the avian influenza virus A/Tky/5092/91 (H5N1) were expressed to allow formation of the whole trimeric polymerase complex. To ascertain the specificity of the interaction, we carried out competition assays using increasing amounts of untagged PB1 or chANP32A to displace the luciferase-tagged proteins. Addition of increasing amounts of PB1 or chANP32A decreased the signal between PB1_luc1_ and chANP32A_luc2_ in a dose-dependent manner (Fig. 1d and e). We confirmed that the tagged constructs retained function using a minigenome assay. The polymerase activity measured with tagged constructs decreased in comparison with untagged proteins; however, polymerase activity was still readily detected (Fig. 1f).

### The interaction of chANP32A with the polymerase is greater than that of huANP32A.

**FIG 2:**
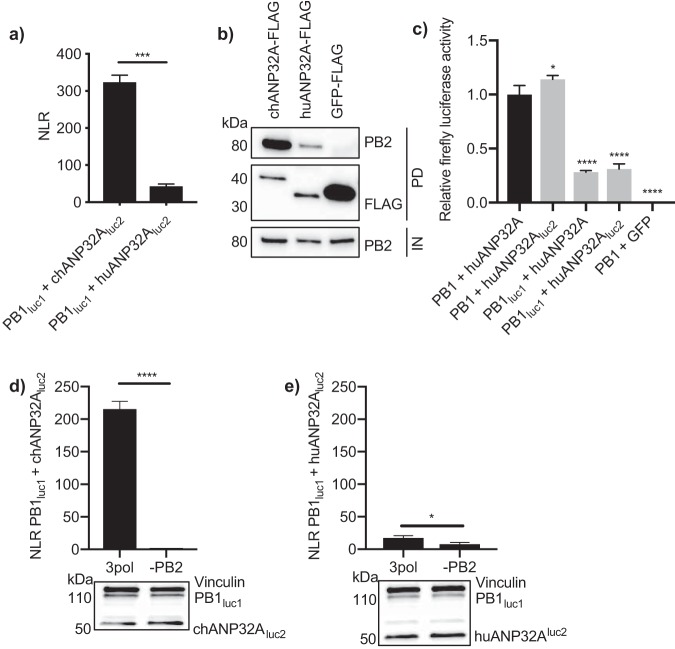
Interaction with the polymerase is greater with chANP32A than with huANP32A. (a) PB1_luc1_, PB2 627E, PA, and either chANP32A_luc2_ or huANP32A_luc2_ were expressed in HEK 293T cells. Twenty-four hours after transfection, cells were lysed, and luminescence was measured. Results shown are mean ± standard deviation from triplicate samples. (b) Expression plasmids encoding PB1, PB2 627E, PA, and either chANP32A-FLAG, huANP32A-FLAG, or GFP-FLAG were transfected into HEK 293T cells. Thirty hours after transfection, cells were lysed, FLAG-tagged proteins were immunoprecipitated, and coprecipitation of PB2 627E was detected using Western blotting. IN, input; PD, pulldown. (c) Minigenome components were transfected into eHAP1 cells in which huANP32A and -B had been knocked out. Conditions included either untagged PB1 or PB1_luc1_ and untagged huANP32A or huANP32A_luc2_. huANP32A was replaced with GFP for use as a negative control. PB2 627K was used under all conditions. Total DNA was kept constant between samples by addition of an empty vector. Results are represented relative to untagged constructs (left black bar). (d and e) PB1_luc1_, PA, and either chANP32A_luc2_ (d) or huANP32A_luc2_ (e) were expressed with the addition or absence of PB2 627E. Twenty-four hours after transfection, cells were lysed, and luminescence was measured. Western blot assay to show expression of tagged plasmids is shown beneath. Vinculin was used as a loading control. *Gaussia* luciferase antibody recognizes both luc1 and luc2. Statistical significance was assessed by Student’s *t* test (*, *P* < 0.05; ***, *P* < 0.001). Results representative of three independent experiments.

The signal from the luciferase complementation assay was at least 7.5 times higher between PB1_luc1_ and chANP32A_luc2_ than with huANP32A_luc2_ (Fig. 2a). To confirm that this difference in interaction was not due to steric differences, for example, the shorter huANP32A protein hindering luciferase reconstitution, we carried out coimmunoprecipitation experiments between chANP32A-FLAG or huANP32A-FLAG and the untagged influenza virus polymerase. Results showed greater coprecipitation of PB2 with chANP32A-FLAG than with huANP32A-FLAG (Fig. 2b). To assess the effect of tagging huANP32A with luc2 on polymerase function, we carried out a minigenome assay in eHAP1 cells lacking huANP32A and -B (11). Tagging huANP32A did not decrease its ability to support polymerase activity; however, tagging PB1 negatively affected polymerase activity in these cells (Fig. 2c). Previous studies have shown that the interaction between ANP32A and the polymerase requires all three subunits of the influenza virus polymerase complex (10, 15). To confirm this, split luciferase complementation assays were carried out in the absence of PB2. Without PB2, the interaction between PB1 and either chANP32A or huANP32A was significantly decreased (Fig. 2d and e). This indicates that ANP32A proteins interact most favorably with the trimeric polymerase complex.

### Interaction between influenza virus polymerase and ANP32A proteins occurs in the nucleus.

**FIG 3:**
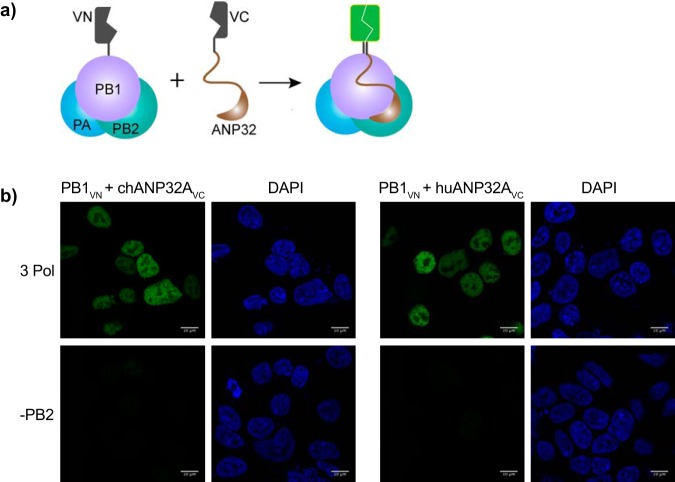
Interactions between the polymerase and ANP32 are localized in the nucleus. (a) Schematic of BiFC assay. Interaction of putative binding partners reconstitutes Venus, resulting in fluorescence. (b) HEK 293T cells were transfected with expression plasmids encoding PB1_VN_, PA, and either chANP32A_VC_ or huANP32A_VC_ in the presence or absence of PB2 627E. Twenty-four hours after transfection, cells were fixed and fluorescence was measured by confocal microscopy. Bars, 10 μm.

The above-described experiments as well as those in previously published studies (10, 12 – 16) measured interactions between ANP32 proteins and influenza virus polymerase in cell lysates. In order to visualize these interactions *in situ*, we carried out bimolecular fluorescence complementation (BiFC) assays (17). The N terminus of Venus fluorescent protein was fused to the C terminus of PB1, and the C terminus of Venus was fused to the C terminus of chANP32A or huANP32A (Fig. 3a). Fluorescence was observed only when trimeric polymerase was present. The fluorescence was localized in the cell nuclei, indicating that this is the site of interaction between the polymerase and ANP32A (Fig. 3b).

### Greater binding of chANP32A to influenza virus polymerase depends on the presence of the PB2 627 domain.

**FIG 4:**
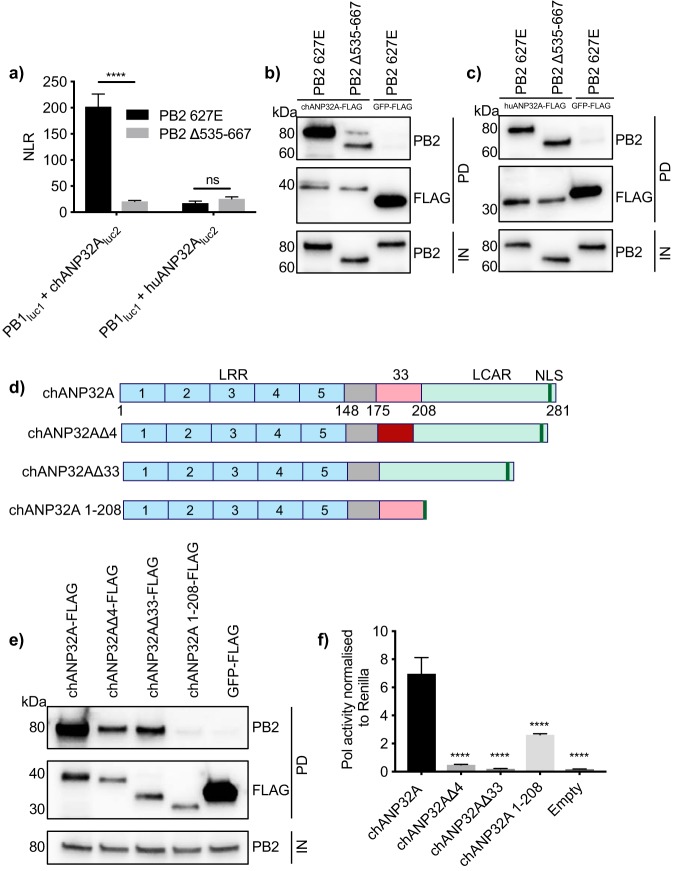
Interactions require the 627 domain of PB2 and the LCAR domain of chANP32A. (a) HEK 293T cells were transfected with expression plasmids encoding PB1_luc1_, PA, either chANP32A_luc2_ or huANP32A_luc2_, and either PB2 627E or PB2 Δ535–667. Twenty-four hours after transfection, cells were lysed, and luminescence was measured. Statistical significance was assessed by multiple *t* tests. (b and c) chANP32A-FLAG (b) or huANP32A-FLAG (c) was expressed in HEK 293T cells with PB1, PA, and either PB2 627E or PB2 Δ535–667. GFP-FLAG was used as a control in place of ANP32-FLAG. Thirty hours after transfection, cell were lysed, FLAG-tagged proteins were immunoprecipitated, and coprecipitation of PB2 was detected using Western blotting. IN, input; PD, pulldown. (d) Schematic of chANP32A mutants. NLS, nuclear localization signal. (e) HEK 293T cells were transfected with expression plasmids encoding PB1, PB2, PA, and either chANP32A-FLAG, chANP32AΔ4-FLAG, chANP32AΔ33-FLAG, chANP32A 1–208-FLAG, or GFP-FLAG. Thirty hours after transfection, cells were lysed, FLAG-tagged proteins were immunoprecipitated, and coprecipitation of PB2 was detected using Western blotting. (f) HEK 293T cells were transfected with expression plasmids encoding the heterotrimeric polymerase subunits and NP and a PolI plasmid expressing an influenza virus-like RNA as well as either chANP32A-FLAG, chANP32AΔ4-FLAG, chANP32AΔ33-FLAG, chANP32A 1–208-FLAG, or an empty plasmid. Expression of *Renilla* luciferase was used as an internal control. Twenty-four hours after transfection, cells were lysed, and luminescence was measured. Results shown are mean ± standard deviation from triplicate samples. Results representative of three independent experiments. Statistical significance was assessed by one-way analysis of variance, and comparisons were made to chANP32A (black bar). ns, nonsignificant; ***, *P* < 0.001; ****, *P* < 0.0001.

We next sought to investigate which domains of the influenza virus polymerase were responsible for its strong interaction with chANP32A. Since the 627 domain of PB2 is implicated in host range, we investigated whether removal of the 627 domain (PB2 Δ535–667) affected binding of chANP32A or huANP32A. It has previously been shown that deletion of these amino acids from PB2 does not prevent formation of a heterotrimeric polymerase complex (18). Results of both split luciferase and coimmunoprecipitation experiments showed that removal of the 627 domain of PB2 resulted in a significant decrease in interaction between the polymerase and chANP32A (Fig. 4a and b). However, removal of the PB2 627 domain did not affect the interaction between huANP32A and the polymerase (Fig. 4a and c). Since chANP32A harbors a 33-amino-acid insertion which is not present in huANP32A, it is likely that the interaction involving the 627 domain of PB2 involves these 33 amino acids.

To look further into which part of chANP32A is important for binding to the avian-origin influenza virus polymerase, we created a series of deletions in the gene (Fig. 4d). Removal of the 33-amino-acid insertion (chANP32AΔ33) reduced binding to polymerase (Fig. 4e). It has previously been shown that the first six of these 33 amino acids form part of a SUMO-interacting motif (SIM), which is important for the ability of chANP32A to support avian viral polymerase (9). We found that removal of the first 4 amino acids (chANP32AΔ4) decreased binding of chANP32A to the polymerase. To test the effect of these mutants on polymerase function, a minigenome assay was carried out in human 293T cells. Removal of the SIM (chANP32AΔ4) reduced the ability to support avian-origin (PB2 627E) polymerase activity 14-fold (Fig. 4f). Removal of the full 33 amino acids (chANP32AΔ33) completely prevented chANP32A from supporting the activity of the avian polymerase (Fig. 4f). However, deletion of the entire LCAR domain (chANP32A 1–208) almost completely abrogated binding, while its ability to support polymerase function decreased only 3-fold. (Fig. 4e and f). Altogether, this indicates that, although required for maximal polymerase activity, binding between chANP32A and avian-origin viral polymerase does not directly correlate with the ability of chANP32A to support polymerase function. The LCAR can stabilize the interaction between chANP32A and polymerase, while the additional 33 amino acids and in particular the presence of 4 amino acids at the putative SIM, present in chANP32A and absent in huANP32 proteins, are required to support the function of the avian virus polymerase.

### Interactions between ANP32A and polymerase-bearing PB2 E627K do not explain why huANP32A can support human-adapted viral polymerase.

**FIG 5:**
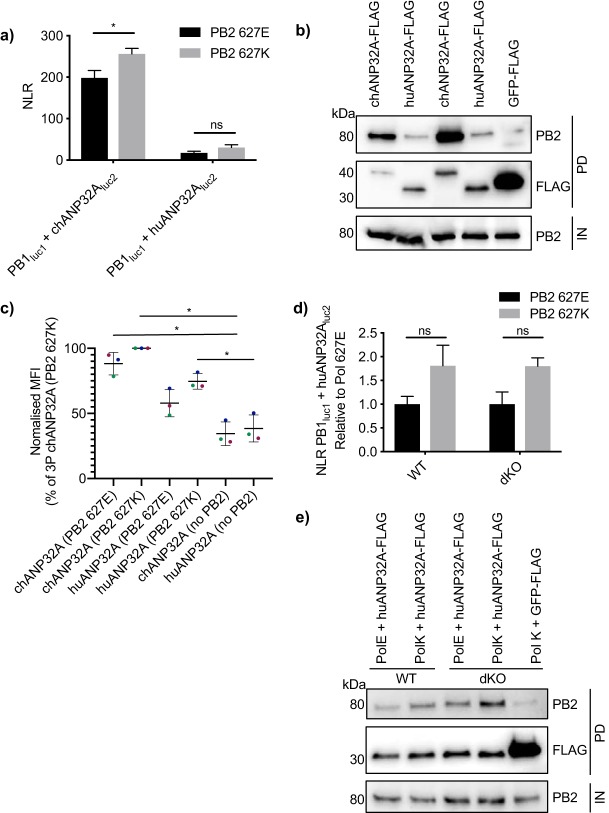
The PB2 E627K mutation does not significantly increase interaction with huANP32A. (a) Expression plasmids encoding chANP32A_luc2_ or huANP32A_luc2_ were transfected into HEK 293T cells with PB1_luc1_, PA, and either PB2 627E (black bars) or PB2 627K (gray bars). Twenty-four hours after transfection, cells were lysed, and luminescence was measured. Statistical significance was assessed by multiple *t* tests and comparisons between PB2 627E and PB2 627K were made. (b) chANP32A-FLAG or huANP32A-FLAG was expressed in HEK 293T cells with PB1, PA, and either PB2 627E or PB2 627K. Thirty hours after transfection, cells were lysed, FLAG-tagged proteins were immunoprecipitated, and coprecipitation of PB2 was detected using Western blotting. IN, input; PD, pulldown. (c) HEK 293T cells were transfected with expression plasmids encoding PB1_VN_, PA, either PB2 627E or PB2 627K, and either chANP32A_VC_ or huANP32A_VC_. Twenty-four hours after transfection, cells were fixed and mean fluorescence intensity (MFI) was quantified by flow cytometry. Different colors represent three independent experiments. Statistical significance was assessed using one-way analysis of variance. *, *P* < 0.05. (d) eHAP1 control (WT) or double-knockout (dKO) cells were transfected with expression plasmids encoding PB1_luc1_, PA, huANP32A_luc2_, and either PB2 627E (black bars) or PB2 627K (gray bars). Twenty-four hours after transfection, cells were lysed, and luminescence was measured. Results presented relative to luminescence from 627E-containing samples. (e) eHAP1 WT or dKO cells were transfected with expression plasmids encoding PB1, PB2 627E or PB2 627K, PA, and either huANP32A-FLAG or GFP-FLAG. Thirty hours after transfection, cells were lysed, FLAG-tagged proteins were immunoprecipitated, and coprecipitation of PB2 was detected using Western blotting. Statistical significance was assessed by two-way analysis of variance, and comparisons were made between PB2 627E and 627K. ns, nonsignificant; *, *P* < 0.05. Results representative of three independent experiments.

chANP32A is able to support the activity of an avian-origin influenza virus polymerase, whereas huANP32A cannot (8). huANP32A and -B can support the activity of human-adapted influenza virus polymerases (10 – 13). As mentioned, the most common humanizing mutation in the polymerase that permits polymerase activity in mammalian cells is the PB2 E627K mutation. We therefore examined whether the interaction between huANP32A and the polymerase increased when the polymerase contained PB2 bearing the E627K mutation. Results of both split luciferase and coimmunoprecipitation assays revealed that interaction of both chANP32A and huANP32A was slightly greater with polymerase containing PB2 627K than with that containing PB2 627E (Fig. 5a and b). However, the increase in interaction of huANP32A with the 627K polymerase was not statistically significant and did not reach levels of chANP32A binding. The same trend could be seen by measuring mean fluorescent intensity of BiFC fusion constructs with polymerases containing either PB2 627E or PB2 627K (Fig. 5c). To ensure that endogenous huANP32A and huANP32B were not masking the binding capacity of PB2 627K for the tagged huANP32A, we also measured interactions between the polymerase and huANP32A in eHAP1 cells where both huANP32A and huANP32B have been knocked out by CRISPR-Cas9 (11). In these double-knockout (dKO) cells, the small increase in interaction between huANP32A and the polymerase containing PB2 627K compared to that with the polymerase containing PB2 627E was still not significant, nor was the difference enhanced compared to control cells (wild type [WT]) that still expressed endogenous huANP32 proteins (Fig. 5d and e). Together, this suggests that increase in the strength of interaction alone is unlikely to explain why the human-adapted polymerase with PB2 627K is able to utilize huANP32A, where the avian-origin polymerase with PB2 627E cannot.

### Interaction of ANP32A proteins with influenza virus polymerase is enhanced in the presence of nonreplicated viral RNA but decreased when polymerase is active.

**FIG 6:**
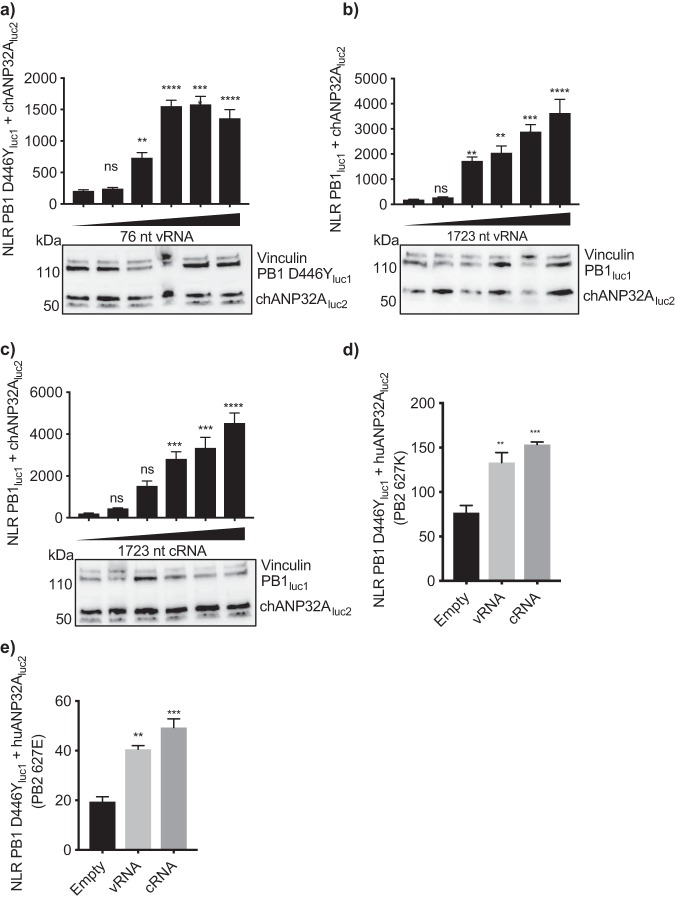
The ANP32-polymerase interaction is stabilized at RNPs. (a to c) HEK 293T cells were transfected with expression plasmids encoding PB2 627E, PA, chANP32A_luc2_, PB1 D446Y_luc1_ (a) or PB1_luc1_ (b and c), and 0, 10, 100, 200, 300, or 400 ng of either a PolI plasmid expressing an influenza virus-like vRNA of 76 nt in length (a), a PolI plasmid expressing an influenza virus-like vRNA of 1,723 nt in length (b), or a PolI plasmid expressing an influenza virus-like cRNA of 1,723 nt in length (c). Twenty-four hours after transfection, cells were lysed, and luminescence was measured. Western blotting assay to show expression of tagged plasmids is shown beneath the graphs. Vinculin was used as a loading control. *Gaussia* luciferase antibody recognizes both luc1 and luc2. (d and e) Cells were transfected with PB1 D446Y_luc1_, huANP32A_luc2_, PA, 400 ng of a PolI plasmid expressing a 1,723-nt vRNA or cRNA, and either PB2 627K (d) or PB2 627E (e). Statistical significance was assessed by one-way analysis of variance, and comparisons were made to sample with no added RNA (left black bar). ns, nonsignificant; **, *P* < 0.01; ***, *P* < 0.001; ****, *P* < 0.0001. Results representative of three independent experiments.

The above binding experiments measured the interaction between ANP32A and trimeric polymerase (apoenzyme); however, the active vRNP complex also contains a viral-like RNA and NP. Influenza virus polymerase has a dynamic structure which can undergo conformational changes when bound to the viral RNA promoter sequences (19 – 22). Specifically, the N terminus of PA and C-terminal domains, including the 627 domain, of PB2 undergo major shifts upon RNA binding (19). We therefore investigated whether the addition of a viral-like RNA affected the interaction between an inactive polymerase and ANP32A, by conducting split luciferase complementation assays in the presence of a PolI plasmid expressing a viral-like RNA 76 nucleotides (nt) in length. Since the polymerase is able to replicate RNA up to 76 nt in length in the absence of NP (23), we constructed a luc1-tagged PB1 construct with a D446Y mutation in its SDD motif, which renders the PB1 catalytically inactive (24). Addition of increasing amounts of 76-nt RNA resulted in an increase in binding between PB1 D446Y_luc1_ and chANP32A_luc2_ (Fig. 6a). The same result could be seen using a 1,723-nt PolI plasmid which expressed either a negative- or positive-sense influenza virus-like RNA. The latter experiments were performed in the absence of NP to prevent replication (Fig. 6b and c). The interaction between huANP32A and influenza virus polymerase was similarly altered in the presence of viral RNA. Addition of 1,723-nt RNA increased the binding between inactive polymerase (PB1D446Y_luc1_) and huANP32A_luc2_ regardless of the identity of residue 627 of PB2 (Fig. 6d and e).

**FIG 7:**
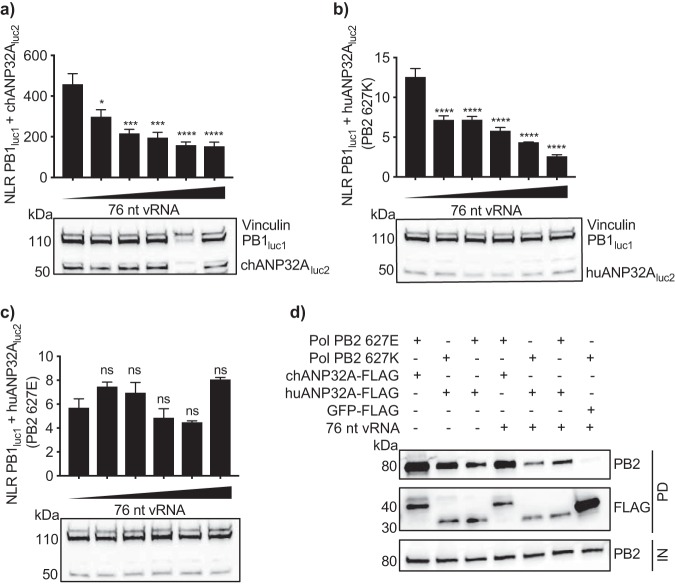
The interaction between ANP32A and polymerase subsides on an active polymerase. (a to c) HEK 293T cells were transfected with PB1_luc1_; PA; 0, 0.625, 1.25, 2.5, 5, or 10 ng PolI 76-nt vRNA; and either chANP32A_luc2_ and PB2 627E (a), huANP32A_luc2_ and PB2 627K (b), or huANP32A_luc2_ and PB2 627E (c). Western blotting assay to show expression of tagged plasmids is shown beneath the graphs. Vinculin was used as a loading control. *Gaussia* luciferase antibody recognizes both luc1 and luc2. Statistical significance was assessed by one-way analysis of variance, and comparisons were made to sample with no added RNA (leftmost bar). ns, nonsignificant; *, *P* < 0.05; **, *P* < 0.01; ***, *P* < 0.001; ****, *P* < 0.0001. (d) HEK 293T cells were transfected with expression plasmids encoding PB1, PB2 627E or PB2 627K, PA, and either chANP32A-FLAG or huANP32A-FLAG in the presence or absence of a PolI plasmid expressing a viral-like RNA. GFP-FLAG was used as a control. Thirty hours after transfection, cells were lysed, FLAG-tagged proteins were immunoprecipitated, and coprecipitation of PB2 was detected using Western blotting. IN, input; PD, pulldown. Results representative of three independent experiments.

Interestingly, using a catalytically active polymerase resulted in the converse effect—we saw a loss of binding between PB1_luc1_ and chANP32A_luc2_ when provided with the 76-nt vRNA template (Fig. 7a). The addition of a viral-like RNA also decreased the interaction with huANP32A if the polymerase contained PB2 627K (Fig. 7b). However, this was not the case for the active PB2 627E polymerase; here, the interaction with huANP32A remained intact in the presence of the 76-nt RNA (Fig. 7c).

To ensure that this reduction in signal was not a consequence of conformational changes in the polymerase sterically hindering luciferase reconstitution, a coimmunoprecipitation assay in the presence or absence of the 76-nt RNA was carried out. A decrease in coprecipitation of PB2 627E with chANP32A and of PB2 627K with huANP32A was observed, whereas coprecipitation of PB2 627E with huANP32A was largely unaffected by the presence of vRNA. The loss of interaction indicates that ANP32A does not interact with the conformation of catalytically active polymerase (Fig. 7d).

### Mutations in the viral promoter do not overcome the requirement for ANP32 for polymerase activity.

**FIG 8:**
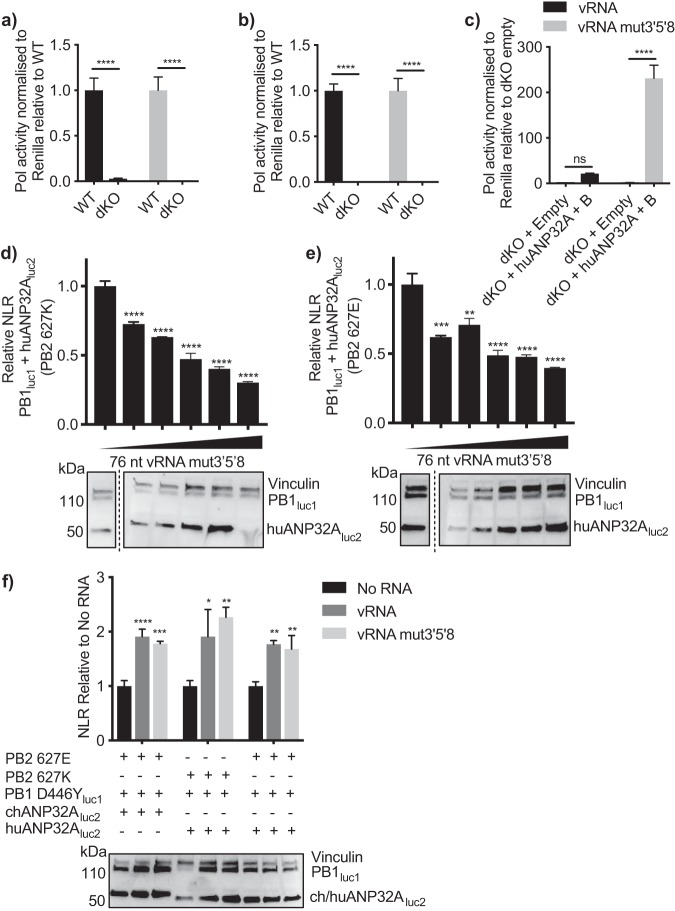
huANP32A is still required for replication of a vRNA template with 3′5′8 mutations. (a and b) WT or dKO eHAP1 cells were transfected with minigenome assay components containing a polymerase bearing either PB2 627E (a) or PB2 627K (b) and PolI plasmids expressing either wild-type vRNA (black bars) or with the 3′5′8 mutations (gray bars). Results were normalized to *Renilla* luciferase and presented relative to control cells (WT). (c) eHAP1 dKO cells were transfected with minigenome assay components using only polymerase containing PB2 627E. Either 40 ng empty plasmid or 20 ng huANP32A and 20 ng huANP32B were also expressed. Results were normalized to *Renilla* luciferase and presented relative to dKO empty plasmid. Statistical significance was assessed by multiple *t* tests. ns, nonsignificant; **, *P* < 0.01; ****, *P* < 0.0001. (d and e) 293T cells were transfected with either PB2 627K (d) or PB2 627E (e) and PB1_luc1_, PA, and 0, 0.625, 1.25, 2.5, 5, or 10 ng PolI 76-nt vRNA. (f) Cells were transfected with PB1 D446Y_luc1_, ANP32A_luc2_, PA, and either no RNA, 400 ng PolI 76-nt vRNA, or 76-nt vRNA mut3′5′8. Western blotting assay to show expression of tagged plasmids is shown beneath the graphs. Vinculin was used as a loading control. *Gaussia* luciferase antibody recognizes both luc1 and luc2. Statistical significance was assessed by one-way analysis of variance, and comparisons were made to sample with no added RNA (black bars). *, *P* < 0.05; **, *P* < 0.01; ***, *P* < 0.001; ****, *P* < 0.0001. Results representative of three independent experiments.

Previous studies have shown that avian-origin influenza polymerase can replicate an influenza viral-type RNA in human cells if the RNA contains G→A, U→C, and C→U mutations at positions 3, 5, and 8, respectively, of the 3′ promoter of vRNA (25). Baker et al. previously showed that exogenous expression of chANP32A in human cells could not further increase the activity of an avian polymerase when the vRNA contained these mutations (15). One interpretation of this result could be that the promoter mutations abrogate the requirement for ANP32A to support influenza virus polymerase. To further investigate this, a minigenome assay was carried out using either an influenza virus-like wild-type vRNA or one harboring the 3′5′8 mutations, in eHAP1 cells lacking huANP32A or -B (11). In the absence of huANP32A or -B, neither an avian-origin nor a humanized polymerase was capable of replicating either viral-like RNA, even that which contained the promoter mutations (Fig. 8a and b). Expression of exogenous huANP32A and -B rescued the ability of the avian-origin polymerase to replicate the vRNA containing the 3′5′8 mutations to a greater extent than wild-type vRNA (Fig. 8c). This demonstrates that, even though these mutations in the promoter sequence can overcome the host restriction of the avian-origin polymerase, ANP32 proteins are still absolutely required to support polymerase activity.

Finally, bearing in mind that avian-origin polymerase was able to replicate an RNA with promoter mutations but not wild-type RNA in the presence of huANP32A, we tested whether addition of the RNA with promoter mutations affected the interaction between polymerase and host factor. The addition of the mutated RNA decreased the interaction between the active polymerase bearing either 627E or 627K and huANP32A (Fig. 8d and e), whereas, as seen in Fig. 7b and c, the addition of wild-type RNA could decrease only interactions involving the PB2 627K polymerase. As in Fig. 6 with wild-type RNA, addition of mutated RNA increased the interaction between ANP32A proteins and an inactive polymerase (Fig. 8f).

Altogether, this suggests that the interaction between influenza virus polymerase and host factor ANP32 is decreased when the polymerase is actively replicating a template RNA.

## DISCUSSION

Here, we have used a split luciferase assay to quantify interactions between ANP32A proteins and the IAV polymerase. We show greater binding of chANP32A than huANP32A with the IAV polymerase and demonstrate that this is due to interactions involving the 33 amino acids in chANP32A and the 627 domain of PB2. We show that the interactions between ANP32A proteins and inactive IAV polymerase are stabilized when inactive RNPs are formed but subside under conditions in which the polymerase is active.

Previous studies have shown interactions between ANP32A and the IAV polymerase using coimmunoprecipitation assays (9, 10, 13 – 15). We confirmed these results using quantitative methods and further demonstrate that this interaction also occurs *in situ* in the nucleus of host cells.

Although full-length chANP32A was required for optimum IAV polymerase activity, a truncated protein lacking the LCAR domain, which almost completely lost detectable coprecipitation of PB2 with chANP32A, was still able to support some polymerase activity. Removal of the avian host-specific 33 amino acids, on the other hand, had a more substantial effect on function of the polymerase than it did on binding. These results, which have also been demonstrated previously (9), imply that the function of chANP32A to rescue avian-origin polymerase activity in human cells is primarily mediated by the additional amino acids present in avian ANP32A orthologues. The strong interaction of chANP32A with the avian-origin polymerase was mapped to the PB2 627 domain and was slightly enhanced for a polymerase containing PB2 627K over one containing PB2 627E. In contrast, the much weaker interaction of huANP32A with the viral polymerase did not depend on the 627 domain and was not enhanced significantly by the PB2 E627K mutation. Taken together, our data and those of Domingues and Hale (9) and Baker et al. (15) suggest that the interaction between viral polymerase and ANP32 proteins is required but not sufficient for the host factor’s ability to support polymerase activity optimally.

Various structural studies have revealed the viral polymerase in distinct conformations dependent on whether particular viral RNAs were present (19 – 22). Initial studies which showed the polymerase bound to promoter vRNA showed nucleotides 1 to 6 of the 3′ vRNA terminus bound to the polymerase in a “preinitiation” conformation (21). A subsequent study demonstrated the 3′ end within the active site but postulated that additional conformational changes may occur upon transition to an initiation competent conformation (26). Given the dynamic nature of the polymerase structure, it is possible that binding to ANP32A may stabilize a specific conformation required for efficient initiation or promoter binding. Indeed, we saw that the interactions between ANP32A and the polymerase were increased at RNPs that were formed with inactive polymerase. This is in line with a previous study which showed the same pattern with a splice variant of chANP32A, corresponding to the chANP32AΔ4 construct used here (15). Replication of the cRNA template to vRNA products requires a transacting or transactivating polymerase in addition to the polymerase complex resident on the template. Bearing in mind that association with RNA alters the polymerase structure, it is likely that the transacting or transactivating polymerase has a different conformation than the resident one (27, 28). Which of the several polymerase complexes ANP32A interacts with is unclear.

Interestingly, the signal between chANP32A and a PB2 627E polymerase and between huANP32A and a PB2 627K polymerase decreased when the polymerase was active. Sugiyama et al. did not detect an association between ANP32 proteins and RNPs in virus-infected cells, which could be explained if the interaction with polymerase at RNPs is transient and not readily captured during infection (10). However, we found that the interaction between a PB2 627E polymerase and huANP32A, a pairing that does not result in polymerase activity, did not decrease when a vRNA template was provided.

**FIG 9:**
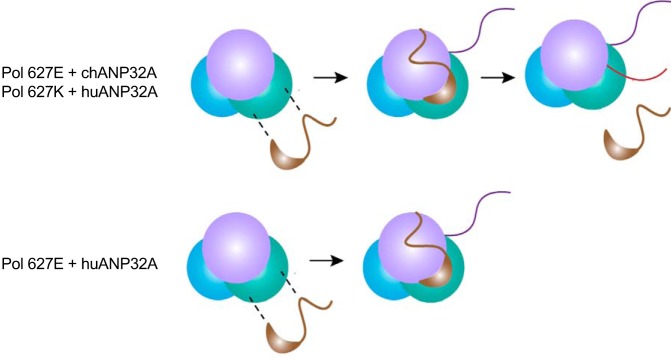
Model of interactions between ANP32A and the viral polymerase. ANP32A can bind to the apopolymerase. This interaction is stabilized at inactive RNPs (violet line represents vRNA), and this is not dependent on species of ANP32A or mutations in the viral polymerase. Active replication (shown by pink line representing newly synthesized cRNA) results in a loss of interaction between ANP32A and viral polymerase. The interaction between huANP32A and an active PB2 627E polymerase does not decrease. Whether this is cause or effect is unclear.

Further evidence to suggest that the restriction in avian-origin polymerases in human cells lies within the interactions involving viral RNA promoter sequence and polymerase is that 3′G→A, 5′U→C, and 8′C→U mutations in the 3′ vRNA promoter are able to overcome this restriction (25, 29). Expression of chANP32A in human cells did not further potentiate the activity of an avian-origin IAV polymerase when this promoter was present (15). We show here that in the absence of ANP32 proteins, the viral polymerase cannot function even on a viral RNA template with these promoter mutations. This suggests that ANP32 proteins are absolutely required for a functional polymerase. Importantly, when the usually inactive pairing of huANP32A and avian-origin polymerase was provided with the promoter-mutated vRNA template, their association decreased as seen for other active polymerase-ANP32 combinations. Taken together, the data suggest that the dissociation of ANP32A with polymerase is a consequence of replication. Alternatively, it could be speculated that the reason that the avian-origin polymerase cannot replicate the wild-type vRNA in the presence of short ANP32 proteins is because huANP32A does not dissociate. Perhaps, transition into a specific conformation triggers release of ANP32A, but incompatibilities between huANP32A and the avian-origin polymerase caused by the glutamic acid residue at position 627 may not allow this transition on wild-type RNA (Fig. 9).

In summary, we have gained further insight into the binding patterns of host factor ANP32A with the IAV polymerase. An understanding of how differences in this protein between species affect its ability to support the viral polymerase contributes to our knowledge of host range, aiding the discovery of new targets for therapeutics for the treatment of influenza.

## MATERIALS AND METHODS

### Cells.

HEK 293T cells (ATCC) were cultured in Dulbecco’s modified Eagle’s medium (DMEM) (Gibco) supplemented with 10% fetal calf serum (FCS) (Biosera), 1% penicillin-streptomycin (Pen-Strep) (Gibco), and 1% nonessential amino acids (Sigma-Aldrich). Human eHAP1 cells (Horizon Discovery) were cultured in Iscove’s modified Dulbecco’s medium (IMDM) (Gibco) supplemented with 10% FCS, 1% Pen-Strep, and 1% nonessential amino acids. Cells were maintained at 37°C and 5% CO_2_.

### Plasmids.

luc1 and luc2 were generated using the *Gaussia* luciferase template, which was synthesized by gene synthesis (GeneArt, ThermoFisher) based on the sequence described by Cassonnet et al. (30). For construction of luc-tagged plasmids, fusion of luc1 or luc2 with polymerase subunits or ANP32 proteins was performed using overlapping PCR, including the linker sequence AAAGGGGSGGGGS. Construction of BiFC constructs was based on that described previously (17). The N or C terminus of Venus was fused onto the C terminus of PB1 or ANP32A, respectively, using overlapping PCR, including the linker sequence GGGGSGGGGS. FLAG-tagged ANP32A constructs have been described previously (8), and chANP32A 1–208 and chANP32AΔ4 were synthesized by gene synthesis (GeneArt, ThermoFisher). PB2 Δ535–667 was generated using overlapping PCR. This construct was generated based on work by Nilsson et al. (18).

### Split luciferase complementation assay.

pCAGGS expression plasmids encoding A/Tky/5092/91 (H5N1) PB1_luc1_, PB2 627E or 627K, PA, and ch- or huANP32A_luc2_ were transfected into 293T or eHAP1 cells at a ratio of 2:2:1:1. Control samples included luc1 and untagged PB1 or luc2 and untagged ANP32A, with all other components remaining constant between all samples (Fig. 1b). Where other polymerase components were luc tagged, equivalent appropriate controls were used.

Twenty-four hours after transfection, cells were lysed in 50 μl (48-well plate) or 100 μl (24-well plate) *Renilla* lysis buffer (Promega) for 1 h at room temperature. *Gaussia* luciferase activity was assayed using the *Renilla* luciferase kit (Promega). Injection of substrate and measurement of bioluminescence were carried out using the FLUOstar Omega plate reader (BMG Labtech). Normalized luminescence ratios were calculated by dividing the signal from the chosen interacting partners by the sum of the two controls as described in the work of Cassonnet et al. (30).

### Minigenome assays.

293T or eHAP1 cells were transfected with pCAGGS expression plasmids encoding 20 ng PB1, 20 ng PB2 627E or 627K, 10 ng PA, and 40 ng NP from A/Tky/5092/91 (H5N1) using Lipofectamine 3000 (Invitrogen). To measure polymerase activity, 20 ng PolI reporter plasmid encoding firefly luciferase flanked by the noncoding regions of influenza virus segment 8 was also transfected. A reporter plasmid with G-A, U-C, and C-U mutations at position 3, 5, and 8, respectively, in the 3′ promoter region was used for the experiment in Fig. 8. Ten nanograms of *Renilla* luciferase was used as an internal control. *Renilla* luciferase was not used for the experiments in Fig. 1f and Fig. 2c since *Renilla* luciferase and *Gaussia* luciferase share the same substrate. Twenty nanograms of the indicated ANP32-FLAG constructs was coexpressed to determine their effect on polymerase activity. Tagged constructs replaced untagged plasmids in the experiments in Fig. 1f and Fig. 2c. Twenty-four hours after transfection, cells were lysed in 50 μl passive lysis buffer (Promega) for 20 min at room temperature. Bioluminescence generated by firefly and *Renilla* luciferase was measured using the dual-luciferase system (Promega) using a FLUOstar Omega plate reader (BMG Labtech).

### Bimolecular fluorescence complementation assay.

293T cells were seeded onto glass-bottomed 8-well chamber μ-slides (Ibidi). pCAGGS expression plasmids encoding 80 ng PB1_VN_, 40 ng PA, 80 ng PB2 627E or PB2 627K, and either 40 ng chANP32A_VC_ or huANP32A_VC_ were transfected into cells using Lipofectamine 3000. Twenty hours after transfection, cells were fixed using 4% paraformaldehyde (Alfa Aesar) for 10 min and permeabilized using 0.2% Triton (Sigma-Aldrich) for 10 min. 4′,6-Diamidino-2-phenylindole (DAPI) staining was used to visualize nuclei. Images were acquired using the Leica SP5 inverted confocal microscope using a Plan-Apochromat 63.0× 1.40-numerical-aperture (NA) objective. Fluorescence was detected using excitation at 480 nm and 514.5 nm with 420- to 480-nm and 525- to 650-nm emission bandwidths for DAPI and Venus, respectively. Images were processed using FIJI software.

### Flow cytometry.

293T cells were transfected as described above for BiFC assays. After resuspension, cells were fixed with 4% paraformaldehyde for 10 min. Following fixation, cells were washed and fluorescence was quantified using the LSRFortessa cell analyzer (BD Biosciences). Cells were gated to include only viable, single cells using side scatter (SSC)-A versus forward scatter (FSC)-A and FSC-H versus FSC-A plots, respectively. Ten thousand events were measured for each sample, and mean fluorescence intensity of yellow fluorescent protein (YFP)-positive cells (480 to 530 nm) was calculated using FlowJo software (version 9).

### Coimmunoprecipitation.

293T or eHAP1 cells were transfected with 3 μg PB1, 3 μg PB2 627E or 627K or PB2 Δ535–667, 1.5 μg PA from A/Tky/5092/91 (H5N1), and 3 μg indicated ANP32-FLAG. Three micrograms of green fluorescent protein (GFP)-FLAG was transfected in place of ANP32-FLAG to be used as a negative control. Seven hundred fifty nanograms of viral-like 76-nucleotide (nt) RNA was used in the experiment in Fig. 7d. Total DNA transfected was kept equal between samples.

Twenty-four hours after transfection, cells were washed in phosphate-buffered saline (PBS) followed by incubation on ice for 20 min in lysis buffer (50 mM Tris-HCl, pH 7.8 [Sigma], 100 mM NaCl, 50 mM KCl, and 0.5% Triton X-100 [Sigma], supplemented with cOmplete EDTA-free protease inhibitor cocktail tablet [Roche]). Samples were centrifuged to pellet cell debris, and cell lysate was collected.

FLAG-tagged proteins were immunoprecipitated using anti-FLAG M2 affinity gel (Sigma-Aldrich). Following three washes at 4°C in Tris-buffered saline (TBS) (Alfa Aesar), cell lysate was incubated with the FLAG affinity gel at 4°C on a rotator overnight. After a further three washes with TBS at 4°C, proteins were eluted by addition of 100 μl 3× FLAG peptide at 150 ng/μl. Coimmunoprecipitated proteins were detected using immunoblot analysis.

### Immunoblot analysis.

All lysates were prepared using the buffer described under “Coimmunoprecipitation.” Lysates were mixed with 2× Laemmli buffer (Sigma-Aldrich) and incubated at 95°C for 10 min to denature proteins. Samples were loaded onto 4 to 20% mini-Protean TXG precast protein gels (Bio-Rad) and transferred onto 0.2-μm polyvinylidene difluoride (PVDF) membranes by semidry transfer (Bio-Rad). Membranes were then blocked in 5% milk for 1 h at room temperature, followed by incubation with appropriate primary antibodies for 1 h at room temperature. Primary antibodies used included rabbit anti-influenza A virus PB2 (GTX125926; GeneTex), mouse anti-FLAG M2 (F1804; Sigma-Aldrich), rabbit anti-*Gaussia* luciferase (E8023; New England BioLabs [NEB]), and rabbit anti-vinculin (EPR8185; Abcam). Following washing in TBS-1% Tween, membranes were incubated with secondary antibodies for 1 h at room temperature. Secondary antibodies included sheep anti-rabbit IgG–horseradish peroxidase (HRP) (AP510P; EMD Millipore) and goat anti-mouse IgG–HRP (STAR11P; Bio-Rad). Following washing, protein bands were detected by chemiluminescence using Amersham ECL prime Western blotting detection reagents (GE Healthcare). Protein bands were imaged using the Fusion Fx imaging system (Vilber Lourmat).
